# A Target-Triggered Emission Enhancement Strategy Based on a Y-Shaped DNA Fluorescent Nanoprobe with Aggregation-Induced Emission Characteristic for microRNA Imaging in Living Cells

**DOI:** 10.3390/molecules28052149

**Published:** 2023-02-24

**Authors:** Zhe Chen, Zhuoyi Wang, Yihua Yuan, Bo Liu, Jiangbo Yu, Zhiwen Wei, Keming Yun

**Affiliations:** 1 School of Forensic Medicine, Shanxi Medical University, Jinzhong 030600, China; 2 Key Laboratory of Forensic Toxicology of Ministry of Public Security, Jinzhong 030600, China; 3 Department of Chemistry, University of Washington, Seattle, WA 98195, USA

**Keywords:** DNA fluorescent nanoprobes, Y-shaped, aggregation-induced emission, microRNA, live-cell imaging

## Abstract

DNA self-assembled fluorescent nanoprobes have been developed for bio-imaging owing to their high resistance to enzyme degradation and great cellular uptake capacity. In this work, we designed a new Y-shaped DNA fluorescent nanoprobe (YFNP) with aggregation-induced emission (AIE) characteristic for microRNA imaging in living cells. With the modification of the AIE dye, the constructed YFNP had a relatively low background fluorescence. However, the YFNP could emit a strong fluorescence due to the generation of microRNA-triggered AIE effect in the presence of target microRNA. Based on the proposed target-triggered emission enhancement strategy, microRNA-21 was detected sensitively and specifically with a detection limit of 122.8 pM. The designed YFNP showed higher bio-stability and cell uptake than the single-stranded DNA fluorescent probe, which has been successfully applied for microRNA imaging in living cells. More importantly, the microRNA-triggered dendrimer structure could be formed after the recognition of target microRNA, achieving a reliable microRNA imaging with a high spatiotemporal resolution. We expect that the proposed YFNP will become a promising candidate for bio-sensing and bio-imaging.

## 1. Introduction

MicroRNAs (miRNAs) are non-coding single-stranded RNA sequences with 19–25 nucleotides in length, whose expression dysregulation is closely related to a variety of human cancers [1,2]. Recently, miRNAs have showed great potential in the early diagnosis and therapy of tumors as clinically important diagnostic and prognostic biomarkers [3]. At present, conventional analysis methods for miRNA detection are mainly based on Northern blotting [4], quantitative reverse transcription polymerase chain reaction (qRT-PCR) [5] and microarray [6]; however, most of them are laborious and not suitable for intracellular applications. Thus, there is an actual need to develop new methods of sensitive analysis and imaging of intracellular miRNAs, which will afford valuable information for understanding the biological processes for the early diagnosis and therapy of tumors.

In recent years, DNA nanotechnology has been demonstrated as a powerful tool for sensing biomarkers in biomedical applications since they possess the advantages of nanometer precision, controllable size, easy programmability and good biocompatibility [7]. So far, a variety of DNA nanostructure-based fluorescent probes have been developed for the sensitive detection of intracellular miRNAs due to their high sensitivity, excellent bio-stability and superior cell uptake efficiency [8,9,10]. For example, Zhang et al. constructed a DNA tetrahedron nanostructure intramolecular catalytic self-assembly platform to detect miRNAs in vitro and live cells via a catalyzed hairpin assembly [11]. Wu et al. developed a rigidified and aptamer targeted DNA triangle-based fluorescent molecular beacon probe for miRNA imaging in living cells with high sensitivity and specificity [12]. Recently, Li et al. designed a DNA nanoprobe based on multivalent self-assembled DNA nanowire for the rapid and tumor-targeted detection of miRNAs [13]. However, most of these DNA nanostructure-based probes needed the use of both a fluorophore and quencher, which not only increases the complexity of the synthesis but also carry a heavy burden for the design of the precise distance and position between the fluorophore and the quencher to generate a detectable signal by fluorescence resonance energy transfer (FRET). Most organic fluorophores used in these fluorescent probes usually suffer from an aggregation-caused quenching (ACQ) effect that their fluorescence will be quenched at high concentrations or in the aggregated state, which will limit their practical applications, as the detection sensitivity may be affected since the probes cannot be used at high concentrations [14]. As the intracellular miRNAs exist in a complex biological environment with low expression levels in live cells [15], therefore, it is still necessary and challenging to develop new fluorescent bio-probes with the advantages of easy fabrication and outstanding photophysical property.

Aggregation-induced emission (AIE) refers to an extraordinary photophysical phenomenon where the fluorophores are non-emissive in good solvents and become highly emissive as aggregates in poor solvents, which was first discovered by Tang’s group in 2001 [16]. The fluorophores with AIE characteristics have opposite properties to the ACQ dyes, including high brightness, large Stokes shift and resistance to photobleaching, which make them ideal signal molecules for the construction of the bio-medical fluorescence probes [17]. Thus, by employing the unique behavior of AIE dyes, a large number of AIE-based fluorescent turn-on probes have been widely designed for bio-analytes detection and imaging [18]. For instance, Xia and co-workers designed water-soluble amphiphilic DNA-AIE probes to detect miRNAs [15,19]. However, both the probes needed the assistance of exonuclease III, which may affect the assay reproducibility and sensing sensitivity, since the optimum activity of the enzyme is usually susceptible to the reaction conditions such as temperature, pH and ionic strength [20,21].

Herein, we developed a new analytical method based on a Y-shaped DNA fluorescent nanoprobe (YFNP) with AIE characteristics to detect intracellular miRNA by combining the merits of AIE dye and DNA nanotechnology. The YFNP is self-assembled by three single-stranded DNA with three AIE dyes modified, which has three extended sticky ends for the recognition of target miRNA from three directions, realizing a high detection sensitivity and specificity. In the presence of target miRNA, after the binding of the two YFNP probes to one target, the miRNA-triggered AIE effect will be induced due to the two AIE dyes are brought into proximity, resulting in a turn-on fluorescence signal. In addition, the resultant Y-shaped YFNP shows advantages of enhanced bio-stability against enzyme degradation and increasing cellular uptake, which is more suitable for intracellular applications and can avoid the false-positive results caused by nuclease digestion. Furthermore, miRNA-triggered dendrimers will be formed after the recognition of the target miRNA, resulting in a further increased fluorescence signal as well as high spatial resolution for the intracellular miRNA imaging. More importantly, it holds a promising potential to be developed into a platform for the simultaneous detection of multiple miRNAs by replacing the target miRNA recognition sequence in the YFNP with recognition sequences for other miRNAs and labeling with multiple AIE dyes. We anticipated that the proposed target-triggered emission enhancement strategy based on the Y-shaped DNA nanoprobe with AIE characteristics will provide a new perspective for designing DNA nanostructure-based nanoprobes for the intracellular applications.

## 2. Results and Discussion

### 2.1. Principle of the YFNP-Based miRNA Assay

**Scheme 1:**
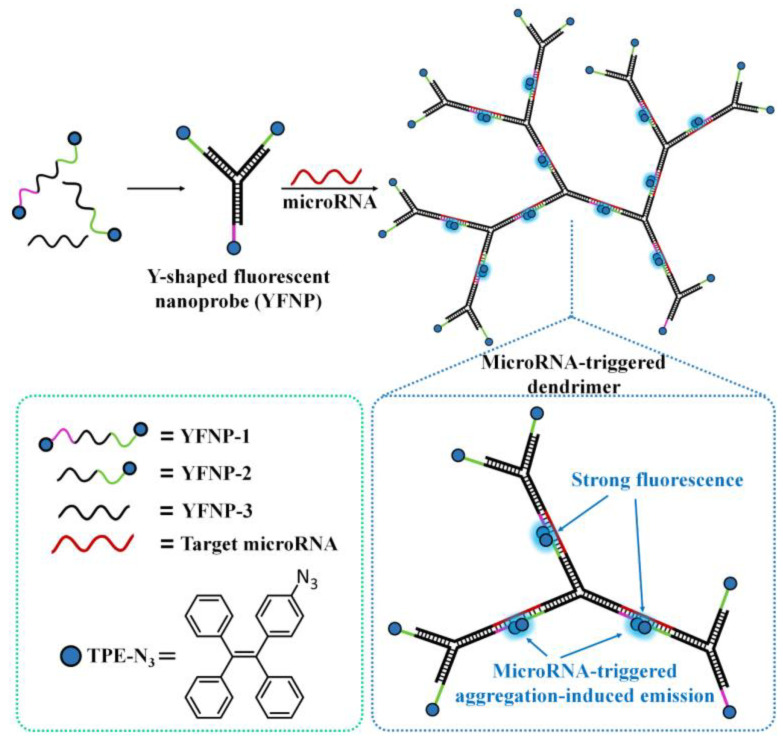
Schematic representation of the YFNP for miRNA detection.

In this work, a Y-shaped DNA fluorescent nanoprobe (YFNP) with AIE characteristics was designed for the intracellular miRNA detection, and the working principle of the proposed target-triggered emission enhancement strategy is depicted in Scheme 1. In particularly, we used the azide-modified tetraphenylethene (TPE-N_3_) as the AIE dye because TPE is easy to be functionalized, which has been widely used for designing bio-probes based on the AIE mechanism [22,23]. Here, microRNA-21 (miR-21) was selected as the target, since it is a biomarker for breast cancer and rectal cancer, which can also regulate the expression of multiple cancer-related genes [24]. As shown in Scheme 1, YFNP was self-assembled by three DNA strands (YFNP-1, YFNP-2 and YFNP-3) based on the basic principle of Watson–Crick base pairing (the detailed sequence information is listed in Table S1). The strand YFNP-1 was chemically labeled with two TPE molecules at both the 5′ and 3′ end; the strand YFNP-2 was chemically labeled with only one TPE at the 3′ end; and there was no chemical labeling of TPE for the strand of YFNP-3. Consequently, the constructed YFNP had three extended sticky ends including two recognition sequences (green color and purple color) that can recognize miR-21 from three directions. As reported, the DNA-TPE conjugates could obtain a satisfactory water solubility by coupling the hydrophilic DNA with hydrophobic TPE, which can result in a low background fluorescence [25,26,27]. It is well known that the major mechanism of AIE effect is based on the restriction of intramolecular motions (RIM), including restriction of intramolecular rotations (RIR) and restriction of intramolecular vibrations (RIV) [28]. In fact, we found that the AIE effect could be induced by just bringing two TPE molecules into close contact (see Figure S1 for more details). Therefore, in the presence of target miR-21, two TPE molecules were brought into close proximity because of the recognition of the YFNP and target, which would induce the RIM process of TPE. Consequently, a miRNA-triggered AIE effect and a turn-on fluorescence would be generated. As a result, the TPE fluorescence intensity observed would be directly proportional to the concentration of miR-21, achieving quantitative detection of the target miR-21. Additionally, miRNA-triggered dendrimers would be further formed after the target miR-21 was recognized by the YFNP, which would further increase the fluorescence signal, making the nanoprobe YFNP ideally suited for the intracellular imaging of miRNA.

### 2.2. Synthesis and Characterizations of the YFNP

**Figure 1:**
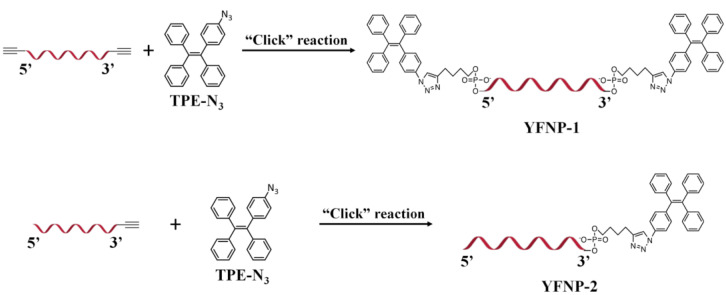
Synthetic route of the strands of YFNP-1 and YFNP-2.

**Figure 2:**
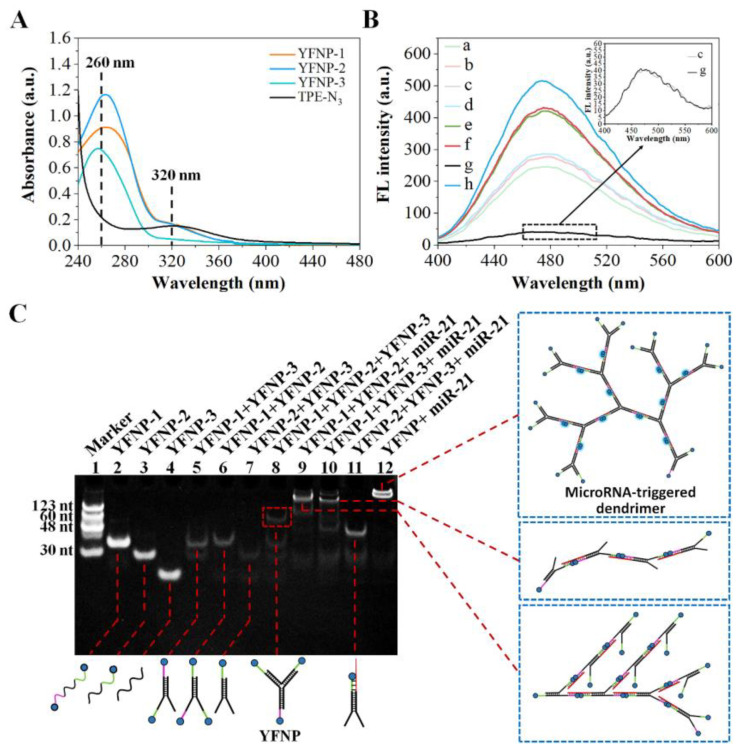
(**A**) UV-Vis absorption spectra of the YFNP-1, YFNP-2, YFNP-3 and TPE-N_3_. TPE-N_3_ = YFNP-2 = YFNP-3 = 5 µM, YFNP-1 = 2.5 µM. (**B**) Study of the feasibility of the assembled YFNP and the double-stranded hybrids in response to the target miR-21. Curve a: YFNP-1+YFNP-3; curve b: YFNP-1+YFNP-2; curve c: YFNP-2+YFNP-3; curve d: YFNP-1+YFNP-2+YFNP-3; curve e: YFNP-1+YFNP-3+miR-21; curve f: YFNP-1+YFNP-2+miR-21; curve g: YFNP-2+YFNP-3+miR-21; curve h: YFNP-1+YFNP-2+YFNP-3+miR-21. Inset image: fluorescence emission spectra of curve c and curve g. (**C**) Characterization by 15% native PAGE. Lane 1: DNA marker. YFNP-1 = YFNP-2 = YFNP-3 = 0.5 µM, miR-21 = 0.1 µM.

The strands of YFNP-1 and YFNP-2 were synthesized via “click” reaction between the azide-modified TPE and alkyne-modified DNA (Figure 1), in which CuBr was utilized as the catalyst based on the previous studies [29,30]. Subsequently, the strands of YFNP-1 and YFNP-2 were purified and collected by the reverse-phase high-performance liquid chromatography (HPLC) (Figure S2). The retention time of the strands was determined according to the absorbance peak at both 260 nm (corresponding to the DNA) and 320 nm (corresponding to the TPE). The synthesized YFNP-1 and YFNP-2 were then characterized and identified by a Q-Exactive electrospray ionization mass spectrometer (ESI-MS) (Figure S3). The molecular weights of YFNP-1 and YFNP-2 measured by ESI-MS were in accordance with the calculated value from their chemical structures, which demonstrated the successful synthesis. As shown in Figure 2A, the successful synthesis of the strands YFNP-1 and YFNP-2 was proved by the co-occurrence of UV absorbance at 260 nm (DNA) and 320 nm (TPE). The YFNP was self-assembled by three strands of YFNP-1, YFNP-2 and YFNP-3, and the structure of the YFNP was further characterized by 15% native polyacrylamide gel electrophoresis (PAGE). As presented in Figure 2C, the bands in lanes 2–4 corresponded to YFNP-1, YFNP-2 and YFNP-3, respectively. It was obvious that the band in lane 8 (YFNP-1+YFNP-2+YFNP-3) migrated slower than the double-stranded hybrids in lanes 5–7 (lane 5: YFNP-1+YFNP-2; lane 6: YFNP-1+YFNP-3; lane 7: YFNP-2+YFNP-3) and the single-stranded strands in lanes 2–4, indicating that YFNP-1, YFNP-2 and YFNP-3 could successfully self-assemble into a Y-shape nanostructure (YFNP).

### 2.3. Feasibility of the YFNP-Based miRNA Assay

The feasibility of the proposed strategy was demonstrated by detecting the fluorescence response signals of the YFNP nanoprobe before and after recognizing the target miR-21. As shown in Figure 2B, the double-stranded hybrids of YFNP-1 and YFNP-3 (curve a), YFNP-1 and YFNP-2 (curve b), YFNP-2 and YFNP-3 (curve c) displayed relatively weak fluorescence since the DNA-TPE conjugates were highly water-soluble. Accordingly, the assembled YFNP (YFNP-1+YFNP-2+YFNP-3) also showed a relatively low emission owing to the fine aqueous solubility (curve d). On the contrary, two TPE molecules were brought into close proximity when the recognition of the target miRNA occurred, resulting in the RIM of phenylene rings of TPE, which can induce the AIE effect and generate a significantly stronger fluorescence signal. Thus, the double-stranded hybrids of YFNP-1 and YFNP-3, YFNP-1 and YFNP-2 could generate an enhanced fluorescence (curve e: YFNP-1+YFNP-3+miR-21; curve f: YFNP-1+YFNP-2+miR-21) and form the corresponding large structures (Figure 2C, lane 9 and lane 10) in the presence of miR-21 since they both had two recognition sequences for the recognition of miRNA. Meanwhile, there was no obvious fluorescence change for the double-stranded hybrid of YFNP-2 and YFNP-3 with the addition of miR-21 (curve g), which was attributed to only one recognition sequences in the hybrid that could not bring the two TPE molecules into close contact. In contrast, the YFNP showed the strongest fluorescence in the presence of miR-21 (curve h), and the fluorescence change of the YFNP was the highest when compared with that of the double-stranded hybrids after the addition of miR-21 (see Figures S4-S6 for more details of discussion of the difference in fluorescence intensity between the YFNP and double-stranded hybrids).

**Figure 3:**
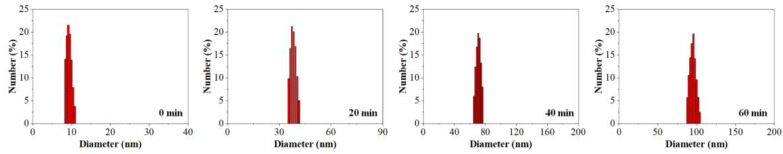
Diameter changes of the YFNP in the presence of target miR-21 at different time intervals (0–60 min).

In addition, the formation of the miRNA-triggered dendrimer of the YFNP was characterized by PAGE (Figure 2C, lane 12). In order to further study the formation of dendrimer structure, dynamic light scattering (DLS) analysis was conducted to monitor diameter changes over time. It was found that the diameter of the YFNP increased from about 9 to 95 nm in the time range of 0 to 60 min in the presence of target miR-21 (Figure 3), demonstrating the formation of the target-triggered dendrimer structure from small to large. In addition, 15% native PAGE was performed to further explore the influence of the concentration of target miRNA for the formation of the dendrimer; as shown in Figure S7, the band brightness of the target-triggered dendrimer was enhanced as the concentration of miR-21 increased in the range of 1–100 nM, which confirmed that the large dendrimer structure was attributed to the binding of the YFNP with the target. Therefore, these results suggest that the proposed YFNP for the detection of miRNA is feasible, as it can generate the strongest AIE effect.

### 2.4. Sensitivity and Selectivity the YFNP-Based miRNA Assay

**Figure 4:**
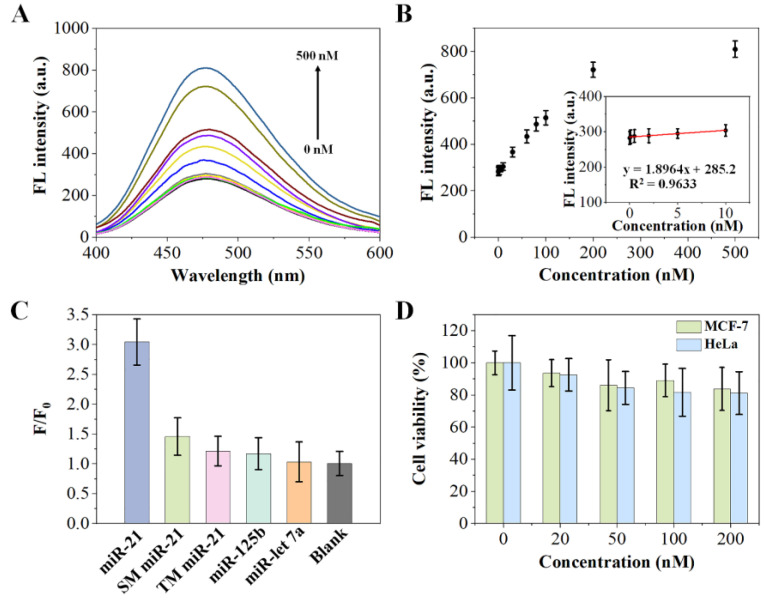
(**A**) Fluorescence response spectra of the YFNP-based assay with different concentrations of miR-21 in the range of 0–500 nM (0, 0.1, 0.5, 2, 5, 10, 30, 60, 80, 100, 200, 500 nM). (**B**) The relationship between the fluorescence intensity of the YFNP at 478 nm and different concentrations of miR-21 in the range of 0 to 500 nM. Inset: the linear plot of the fluorescence intensity at 478 nm versus the concentration of miR-21 (0–10 nM). (**C**) Selectivity assay of responses of the YFNP to miR-21 and its analogues. F was the fluorescence intensity of the YFNP at 478 nm with the addition of different miRNA species, F_0_ was the fluorescence intensity of the YFNP at 478 nm without the addition of the miRNA species. miR-21 = SM miR-21 = TM miR-21 = miR-125b = miR-let 7a = 500 nM. (**D**) The cell viability of MCF-7 cells and HeLa cells after 24 h incubation with different concentrations (20, 50, 100, 200 nM) of the YFNP. Error bars indicate the standard deviation of measurements (n = 3).

To determine the sensitivity of the YFNP-based miRNA assay, the fluorescence spectra of the YFNP were measured in the presence of miR-21 at different concentrations (0, 0.1, 0.5, 2, 5, 10, 30, 60, 80, 100, 200, 500 nM). As shown in Figure 4A,B, as the concentration of the miR-21 increased in the range of 0 to 500 nM, and the YFNP fluorescence was enhanced, which was attributed to the fact that more miR-21 induced the formation of larger miRNA-triggered dendrimer, subsequently resulting in the increase in AIE effect and fluorescence signal. Moreover, it was found that the YFNP fluorescence intensity at 478 nm had a good linear correlation to the concentration of miR-21 in the range of 0 to 10 nM with a correlation coefficient of 0.9633 (inset of Figure 4B), and a limit of detection (LOD) of 122.8 pM was determined according to 3σ/K (σ is the standard deviation and K is the slope of the curve of linear regression). The sensitivity of the proposed fluorescent analytical method based on the YFNP with an AIE characteristic was comparable to some recent miRNA detection approaches (Table S2) [13,31,32,33,34,35,36,37], which might derive from the following factors: (1) the utilization of AIE molecules with special photophysical properties; (2) the increase in fluorescence signal response by the formation of miRNA-triggered dendrimer structures.

In order to demonstrate the quantitative capacity of this method, recovery experiments were conducted by spiking different concentrations of standard miR-21 solutions into a blank detection buffer. Using the standard curve generated from our detection system, the recoveries in the ranges from 96.0 to 109.4% were obtained (Table S3), indicating the good quantitative capacity of the proposed method.

The specificity of the proposed strategy was then evaluated by comparing target miR-21 with its analogues and references, including single-base mismatched miR-21 (SM miR-21), triple-base mismatched miR-21 (TM miR-21), miR-125b and miR-let 7a. As shown in Figure 4C, only the target miR-21 was able to induce the highest fluorescence signal, while the fluorescence intensity of the other miRNAs was comparable to the background fluorescence (blank). These results indicate that the proposed YFNP nanoprobe with AIE property is capable of detecting miRNA with high sensitivity and specificity.

In this work, about 3-fold fluorescence enhancement of the YFNP in the presence of target miRNA was determined, which was attributed to the AIE effect that was induced by the aggregation of only two TPE molecules. Therefore, in the follow-up study, the probe designing with more aggregated TPE molecules will be further explored to induce a stronger AIE effect.

### 2.5. Intracellular miR-21 Imaging

Owing to the high sensitivity and specificity of the YFNP for the detection of miR-21 in vitro, we further explored its application in intracellular miRNA imaging. It has been reported that miR-21 is overexpressed in breast cancer cells; therefore, MCF-7 cells (human breast adenocarcinoma cell line) were selected as the target cells, and HeLa cells (human cervical cancer cell line) were used as a negative control due to their relatively low expression of miR-21 [38,39]. Before detecting the intracellular miRNA, assays of bio-stability and cytotoxicity of the YFNP were conducted, which were crucial properties for live cell studies. We incubated the prepared YFNP with 50% fetal bovine serum (FBS) at 37 °C for 150 min; as shown in Figure S8A, the PAGE results showed that the lane of YFNP still remained a clear band with 150 min incubation, suggesting that the YFNP can keep the Y-shaped nanostructure intact in FBS. Furthermore, the single-stranded YFNP-1 at the same concentration was used as a control. As shown in Figure S8B, the band of YFNP-1 almost disappeared within 150 min. These results indicate that the YFNP has enhanced bio-stability compared with the single-stranded probe, which was likely because of the steric hindrance induced by DNA assembly that will effectively reduce enzyme degradation. More importantly, as compared with the single-stranded fluorophore/quencher-based probe, the enhanced bio-stability of the Y-shaped YFNP can avoid the false-positive signal arising from non-specific enzyme degradation.

We next evaluated the cytotoxicity of the YFNP by MTT (3-(4,5-dimethylthiazol-2-yl)-2,5-diphenyl tetrazolium bromide) assay. MCF-7 and HeLa cells were incubated with different concentrations of the YFNP for 24 h (Figure 4D), and the result of MTT cell viability assay revealed that the YFNP showed low cytotoxicity for both MCF-7 and HeLa cells, indicating the good biocompatibility of the YFNP.

**Figure 5:**
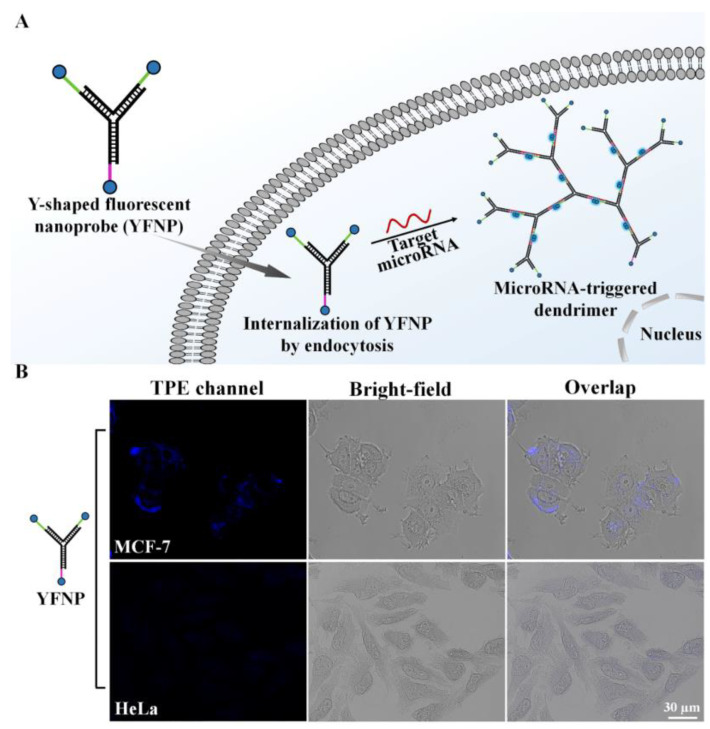
(**A**) Schematic representation of the YFNP for miRNA detection in living cells. (**B**) The confocal fluorescence images of MCF-7 cells and HeLa cells incubated with the YFNP for 6 h.

Subsequently, the capability of the YFNP for intracellular miRNA imaging was investigated by using a laser scanning confocal microscope (LSCM) (Figure 5). MCF-7 cells were treated with the YFNP for 6 h, and the LSCM results showed that the cytoplasm of MCF-7 cells emitted a strong blue fluorescence (Figure 5B), indicating the successful cellular internalization of the YFNP. Meanwhile, HeLa cells were incubated with the YFNP under the same conditions as MCF-7 cells; however, they exhibited negligible fluorescence emission (Figure 5B). The results confirmed that the level of miR-21 expression in MCF-7 cells was higher than that in HeLa cells, which was in accordance with previous reports [30,31].

**Figure 6:**
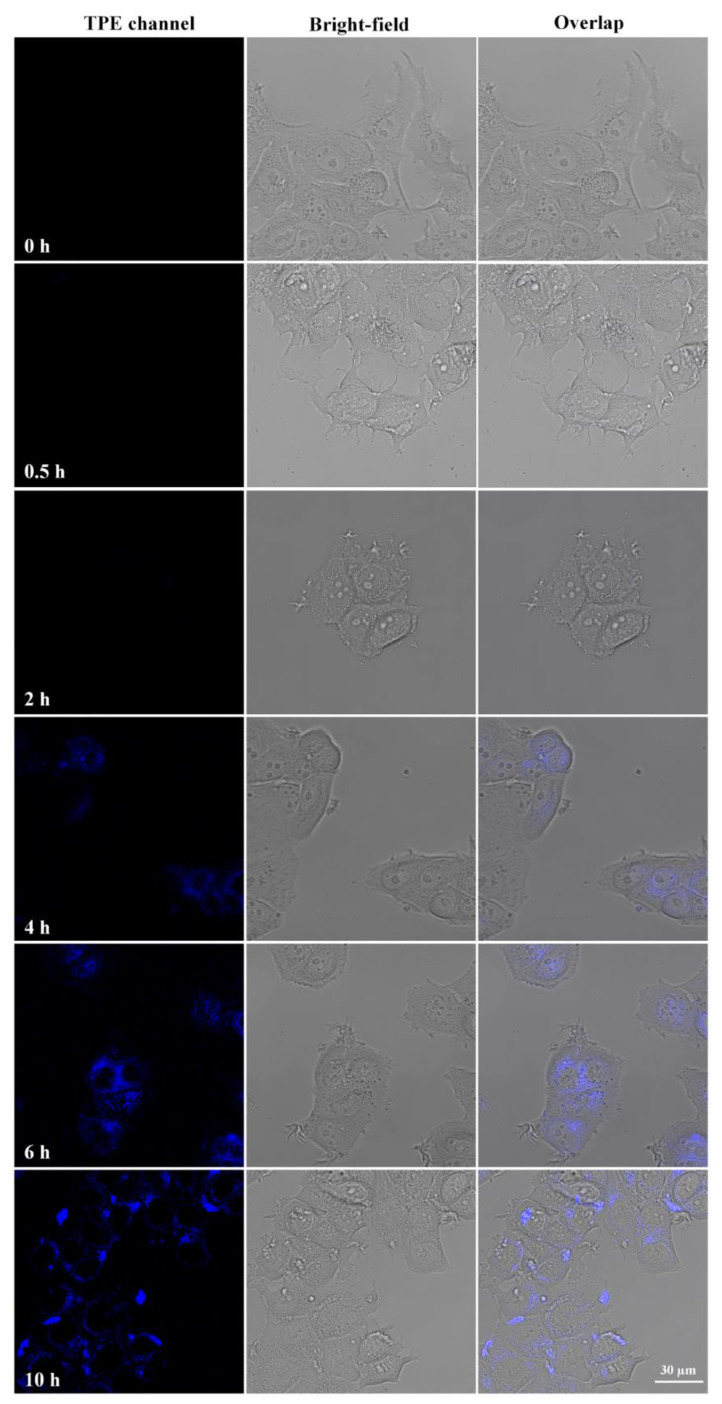
The confocal fluorescence images of MCF-7 cells incubated with the YFNP for different reaction times (0–10 h).

In order to further demonstrate the image ability of YFNP in live cells, MCF-7 cells were treated with the YFNP with different time intervals (0–10 h). As shown in Figure 6, when incubated for 4 h, a weak fluorescence signal was observed in MCF-7 cells as YFNP began to enter the cells. As the incubation time increased, the blue fluorescence signal gradually enhanced. It could be found that the intracellular fluorescence signal was still strong when the incubation time was extended up to 10 h, which further confirmed that the formed large dendrimer structures can enhance the bio-stability of the probe.

**Figure 7:**
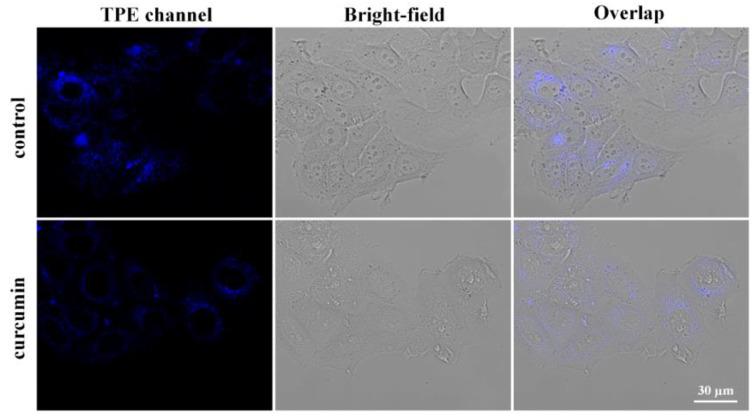
The confocal fluorescence images of MCF-7 cells pretreated with or without curcumin (control), respectively, followed by further incubation with the YFNP (MCF-7 cells were pretreated with curcumin for 24 h).

Curcumin was then introduced to inhibit the expression of intracellular miR-21 to monitor the dynamic expression of miR-21 (Figure 7). A decreased blue fluorescence signal was observed for MCF-7 cells treated with curcumin as compared to the cells treated without the curcumin (control), suggesting that YFNP can monitor the dynamic expression of miRNAs in various cellular states.

Furthermore, cell uptake efficiency of the Y-shaped DNA probe was evaluated (we used the dye of FAM for the label of the sequence to make sure the fluorescence intensity of the Y-shaped probe (YDNA-FAM) and single-stranded probe (SDNA-FAM) is originally identical). As depicted in Figure S9, MCF-7 cells treated with the YDNA-FAM showed an obvious green fluorescence while the SDNA-FAM treated cells exhibited extremely weak fluorescence, demonstrating that the Y-shape DNA assembly shows a higher cell uptake compared with the single-stranded DNA probe, which could be due to the following reasons. (1) The single-stranded YFNP-1 was negatively charged, which was hard to enter cells by passive processes because of the electrostatic repulsion. Similarly, YFNP could not enter cells by passive processes, since the YFNP was also negatively charged. However, the YFNP possessed a Y-shaped nanostructure that could enter cells by endocytosis, which has been proved to have higher cell uptake compared with single-stranded DNA and double-stranded DNA [30,40,41]. (2) The Y-shaped YFNP possessed better bio-stability than the single-stranded YFNP-1 in vivo, which could be uptaken by cells over a longer time period [42].

**Figure 8:**
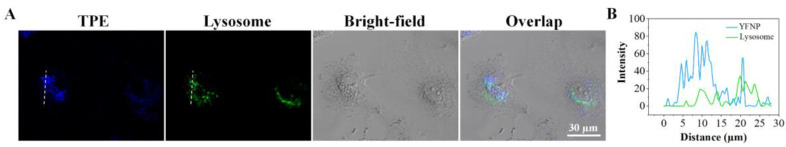
(**A**) The confocal fluorescence images of MCF-7 cells incubated with the YFNP for 6 h and then further incubated with 1 μM Lysotracker Green (30 min). (**B**) The intensity profile of regions of interest (the dotted lines in (**A**)) cross the cells.

Finally, a colocalization experiment was conducted to study the endosomal escape of the YFNP probe. The lysosome was stained with Lysotracker Green; as shown in Figure 8A, the blue fluorescence from YFNP and the green fluorescence from lysosome maker (Lysotracker Green) did not colocalize well in the cytoplasm, indicating that the YFNP successfully escaped from lysosome to recognize target miRNA in the cytoplasm. Additionally, ImageJ software (Version 2.1.4.7) was used to analyze the fluorescence signal intensity of YFNP and lysosome. As shown in Figure 8B, there was little overlap between the YFNP (blue) fluorescence of the probe and the signal of lysosome tracking dye (green), which further indicated that most YFNP have escaped from the lysosomes and subsequently entered into the cytoplasm to detect the target miRNA-21. Moreover, the miRNA-triggered dendrimer structure could be formed in the presence of miR-21, which could not only further increase the intracellular bio-stability but also enhance the fluorescence signal. Thus, YFNP can be applied for reliable miRNA imaging in living cells with a high spatiotemporal resolution.

## 3. Materials and Methods

### 3.1. Materials

All chemicals and solvents were used as received without further purification unless specified otherwise. All the DNA sequences were purified by high-performance liquid chromatography (HPLC) and obtained from Sangon Biotech Co., Ltd. (Shanghai, China). All the RNA sequences were purified by HPLC and ordered from Genewiz Corporation (Nanjing, China). The detailed sequence information is listed in Table S1. N,N,N’,N’-tetramethylethylenediamine (TEMED), acrylamide, bis-acrylamide and 3-(4,5-dimethyl-2-thiazolyl)-2,5-diphenyl-2-H-tetrazolium bromide (MTT) were purchased from Sangon Biotech Co., Ltd. (Shanghai, China). Ammonium persulfate (APS) and 5 × tris/boric acid/EDTA (TBE) buffer were purchased from Bioson Corporation (Beijing, China). The GelRed (10,000×) dye for DNA staining was purchased from Beyotime Biotech Co., Ltd. (Shanghai, China). Copper (I) bromide, tris [(1-benzyl-1H-1,2,3-triazol-4-yl) methyl] amine (TBTA), dimethyl sulfoxide (DMSO), tetrahydrofuran (THF) and dichloromethane (DCM) were obtained from Aladdin Co., Ltd. (Shanghai, China). Fetal bovine serum (FBS), Dulbecco’s Modified Eagle’s Medium (DMEM), penicillin and streptomycin were obtained from Gibco BRL Co., Ltd. (Grand Island, NE, USA). Azide-modified tetraphenylethylene (TPE-N_3_) was synthesized according to the previous procedure [43]. MCD-7 cells and HeLa cells were purchased from the Cell Center of the Institute of Basic Medical Sciences, Chinese Academy of Medical Science.

### 3.2. Apparatus

Fluorescence emission spectra were recorded by an Agilent Cary Eclipse Fluorescence Spectrophotometer (Agilent Co., Santa Clara, CA, USA). UV-vis absorbance spectra were measured via a UV-1800 spectrophotometer (SHIMADZU, Kyoto, Japan). Polyacrylamide gel electrophoresis analysis was carried out on a gel imaging system (Bio-Rad, Hercules, CA, USA). DNA-TPE conjugates were purified via reverse-phase HPLC (Agilent Co., Santa Clara, CA, USA). Dynamic light scattering (DLS) measurements were performed on a Brookhaven NanoBrook Omni instrument (Brookhaven Instruments Corporation, New York, NY, USA). The fluorescence images of cells were obtained from a Leica SP8 confocal microscope (Leica, Wetzlar, Germany).

### 3.3. Synthesis and Purification of the YFNP-1 and YFNP-2

For the synthesis of the strand YFNP-1, 0.1 μmol alkyne-labeled YFNP-1 precursor and 2 μmol TPE-N_3_ were added to a mixture (130 μL) of deionized water and THF (1:1, *v*/*v*). CuBr (1.5 mg, 10 µmol) was dissolved in 90 µL of freshly prepared DMSO:t-butanol (3:1, *v*/*v*) mixture. TBTA (5.3 mg, 10 µmol) was dissolved in 180 µL of freshly prepared DMSO:t-butanol (3:1, *v*/*v*) mixture. CuBr (90 µL) and TBTA (180 µL) were then added to the above mixture. Then, the reaction mixture was stirred for overnight at room temperature under an argon atmosphere. The above reaction solution was subsequently transferred to a 3 K dialysis bag for dialysis. After dialysis of 48 h, the crude products in the dialysis bag were ultrafiltered via 3 K ultra-4 centrifugal filter devices, and 500 µL YFNP-1 concentrate was collected. Lastly, the collected YFNP-1 concentrate was purified via reverse-phase HPLC through a C-18 reverse column using a binary gradient (buffer A: 100 mM triethylammonium acetate (TEAA), buffer B: acetonitrile), and the flow rate was set at 1 mL/min. Similarly, the strand YFNP-2 was synthesized and purified as above.

### 3.4. Preparation of the YFNP

The Y-shaped nanoprobe YFNP was constructed by three strands (YFNP-1, YFNP-2, YFNP-3). YFNP-1, YFNP-2 and YFNP-3 were mixed in the hybridization buffer (10 mM phosphate, 137 mM sodium chloride, 12.5 mM Mg^2+^, pH = 7.4), and the final concentration of each strand was 0.5 µM. The mixture was heated to 95 °C for 5 min and then followed by slowly cooling to the room temperature.

### 3.5. Electrophoresis Characterization

The electrophoresis assay of different DNA structures was investigated by 15% native polyacrylamide gel electrophoresis (PAGE). The gel was run at 100 V for 90 min in 0.5 × TBE buffer (89 mM Tris-borate, 2.0 mM EDTA, 12.5 mM Mg^2+^, pH = 8.3) and stained for 15 min in a 1 × GelRed solution.

### 3.6. In Vitro miRNA Detection

The experiments were performed in 100 μL of the hybridization solution containing 0.1 μM YFNP and different concentrations of target miRNA. Fluorescence emission spectra were recorded with the following settings: the excitation wavelength was 320 nm, and the excitation and emission slits were set for 5.0 and 5.0 nm, respectively. The fluorescence intensity at 400 nm and 600 nm was used for data analysis.

### 3.7. Cell Culture

MCF-7 cells and HeLa cells were cultured in DMEM medium containing 10% FBS, 100 mg/mL penicillin and 100 mg/mL streptomycin in a humidity incubator (5% CO_2_, 37 °C).

### 3.8. MTT Assay

For the evaluation of the cytotoxicity of the proposed nanoprobe YFNP, 200 μL MCF-7 cells and HeLa cells were pre-seeded into a 96-well plate (5000 cells/well) and cultured for 24 h. Then, we removed the medium and the cells were cultured with 200 μL new medium with different concentrations of YFNP (0 μM, 20 μM, 50 μM, 100 μM and 200 μM) for another 24 h. After a wash with 1 × PBS buffer (three times), 100 μL MTT solution (5 mg/mL in PBS) was added to each well and incubated for 3 h. The medium was then removed, and the formazan products were dissolved with 150 μL DMSO. The absorbance of each cell was measured at 490 nm for the calculation of the cell viability.

### 3.9. Intracellular miRNA Imaging

MCF-7 cells and HeLa cells were pre-cultured for 24 h. Then, 200 μL Opti-MEM (reduced-serum medium) containing 200 nM YFNP was added to cells and incubated for 6 h. After the incubation, the cells were washed with 1 × PBS buffer (three times), and the cellular fluorescent images were taken under a Leica SP8 confocal microscope. The laser transmitters were: 405 nm excitation and 450–480 nm emission for TPE.

## 4. Conclusions

In summary, we have developed a Y-shaped DNA nanoprobe with AIE property to detect intracellular miRNA. By integrating with an AIE dye, the resultant YFNP-based sensing system was found to have a reduced background and improved signal-to-noise ratio, resulting in a high detection sensitivity. Moreover, the miRNA-triggered dendrimer structure could be formed in the presence of the target miRNA, which was used as a “small to large” strategy for target-triggered emission enhancement, achieving a high spatial resolution for miRNA imaging. Benefiting from the Y-shaped DNA nanostructure, the YFNP has self-delivery capability, good bio-stability, and low cytotoxicity, which has great potential for the design of DNA-based nanoprobes for early diagnostics of miRNA-related diseases.

## Data Availability

Data are available from the authors upon reasonable request.

## Supplementary Materials

The following supporting information can be downloaded at: https://www.mdpi.com/article/10.3390/molecules28052149/s1, Figure S1: (A) Three types of DNA-TPE conjugates (i–iii) with different conformations. (B) The fluorescence spectra of the three types of DNA-TPE conjugates (i–iii). (C) Comparison of the fluorescence intensity of the DNA-TPE conjugates before and after hybridization: the double-stranded hybrid (iii) showed nearly 1.63-time fluorescence enhancement compared with the sum of the fluorescence intensity of DNA14-3′TPE (i) and cDNA14-5′TPE (ii); Figure S2: Reverse phase-HPLC spectra of YFNP-1 (A) and YFNP-2 (B) with absorbance at both 260 nm (black) and 320 nm (red). The fraction labeled with “*” was collected; Figure S3: Chemical structure and electrospray ionization (ESI) MS spectrum of YFNP-1 (A,C) and YFNP-2 (B,D). YFNP-1: m/z = 2152.3987, z = 6 (12918.12 theoretical); YFNP-2: m/z = 1785.5275, z = 5 (8931.66 theoretical); Figure S4: (A–C) The fluorescence spectra of the single-stranded DNA-TPE conjugates (YFNP-1, YFNP-2 and YFNP-3) and their double-stranded hybrids (YFNP-1 + YFNP-3, YFNP-1 + YFNP-2 and YFNP-2 + YFNP-3) before the addition of target miR-21 in Figure 2B. (D) The AIE effect was evaluated by fluorescence changes before and after the hybridization of the two DNA-TPE conjugates (YFNP-1 + YFNP-3, YFNP-1 + YFNP-2 and YFNP-2 + YFNP-3). F was the fluorescence intensity of the double-stranded hybrids after hybridization of the two DNA-TPE conjugates; F_0_ was the sum of the fluorescence intensity of the two DNA-TPE conjugates before their hybridization; Figure S5: Change of the fluorescence intensity of the assembled YFNP and the double-stranded hybrids (YFNP-1 + YFNP-3, YFNP-1 + YFNP-2 and YFNP-2 + YFNP-3) before and after the addition of target miR-21 in Figure 2B; Figure S6: Schematic representation of the YFNP (A) and the hybrid (YFNP-1+YFNP-2) (B) in response to fully matched target miRNA; Figure S7: 15% native PAGE characterization of the target-triggered dendrimer responding to various concentrations of miRNA-21 in the presence of the YFNP; Figure S8: 10% PAGE analysis of the bio-stability of the self-assembled YFNP (A) and the single-stranded YFNP-1 (B) in the presence of 50% FBS; Figure S9: The confocal fluorescence images of MCF-7 cells incubated with YDNA-FAM and SDNA-FAM, respectively; Table S1: Oligonucleotide sequences used in this work; Table S2: The summary of recent miRNA analytical approaches in comparison with our strategy; Table S3: Results of recovery analysis by the YFNP-based assay.
