# Supplementary material for: A Target-Triggered Emission Enhancement Strategy Based on a Y-Shaped DNA Fluorescent Nanoprobe with Aggregation-Induced Emission Characteristic for microRNA Imaging in Living Cells
Source: Molecules. 2023 Feb 24;28(5):2149. doi: 10.3390/molecules28052149 (PMC10004006; doi:10.3390/molecules28052149)
Supplement: Supplementary file 1 [file molecules-28-02149-s001.zip › molecules-2196282-supplementary.pdf]

Supplementary Materials

A Target-Triggered Emission Enhancement Strategy Based on a Y-Shaped DNA Fluorescent Nanoprobe with Aggregation-Induced Emission Characteristic for microRNA Imaging in Living Cells

Zhe Chen [1,2,†], Zhuoyi Wang [1,2,†], Yihua Yuan [1,2], Bo Liu [1,2], Jiangbo Yu [3,*], Zhiwen Wei [1,2,*], Keming Yun [1,2,*]

[1] School of Forensic Medicine, Shanxi Medical University, Jinzhong, Shanxi 030600, China;

[2] Key Laboratory of Forensic Toxicology of Ministry of Public Security, Shanxi 030600, China;

[3] Department of Chemistry, University of Washington, Seattle, Washington 98195, United States;

[*] Correspondence: jbyu@uw.edu (J.Y.); weizhiwen2000@163.com (Z.W.); yunkeming5142@163.com (K.Y.)

[†] These authors contributed equally to this work.

Table S1. Oligonucleotide sequences used in this work

Strand	Sequence (from 5' to 3')
**YFNP-1**	TPE-CTG ATA AGC TAC GTG CTC AAC TAT GGC TCA ACA TCA GT-TPE
**YFNP-2**	GCC ATA GTG GAT TGC ATC AAC ATC AGT-TPE
**YFNP-3**	TGC AAT CCT GAG CAC G
**miR-21**	UAG CUU AUC AGA CUG AUG UUG A
**Single-base mismatched miR-21 (SM miR-21)**	UAG CUU AUC CGA CUG AUG UUG A
**Triple-base mismatched miR-21 (TM miR-21)**	UAG CUU AUA CCA CUG AUG UUG A
**miR-125b**	UCC CUG AGA CCC UAA CUU GUG A
**miR-let 7a**	UGA GGU AGU AGG UUG UAU AGU U
**miR-NC**	UUG UAC UAC ACA AAA GUA CUG
DNA_{14}-3'TPE	CGT GTG CCT CCA AC-TPE
$cDNA_{14}$-5'TPE	TPE-CGC ACC GCA GTG CG

DNA_{28}	CGC ACT GCG GTG CGG TTG GAG GCA CAC G

Note: The mismatched bases are marked in red.

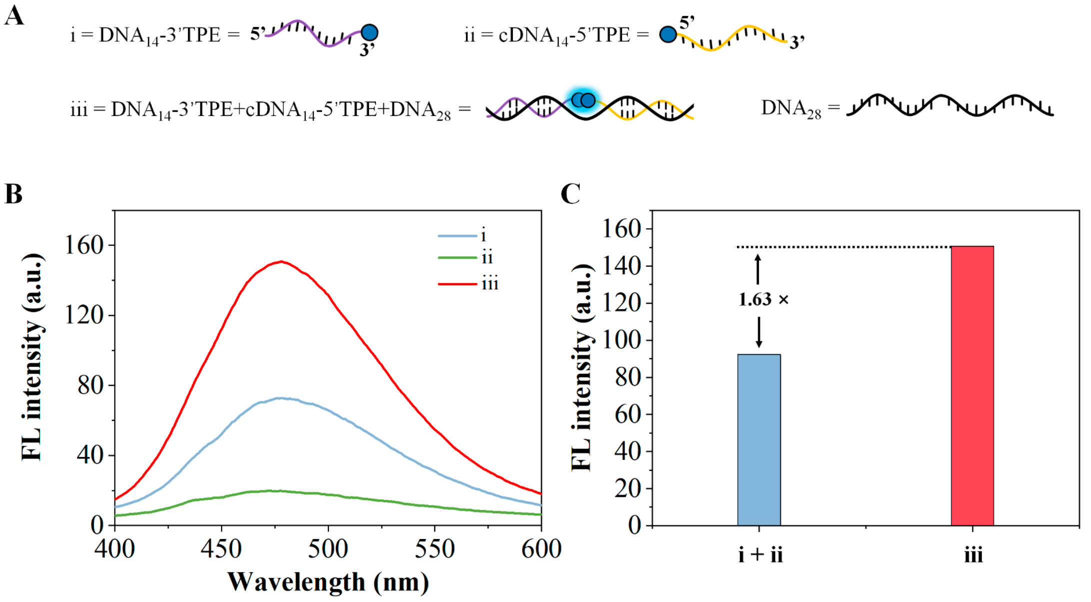

Figure S1. (A) Three types of DNA-TPE conjugates (i-iii) with different conformations. (B) The fluorescence spectra of the three types of DNA-TPE conjugates (i-iii). (C) Comparison of the fluorescence intensity of the DNA-TPE conjugates before and after hybridization: the double-stranded hybrid (iii) showed nearly 1.63-time fluorescence enhancement compared with the sum of the fluorescence intensity of DNA_{14}-3'TPE (i) and $cDNA_{14}$-5'TPE (ii).

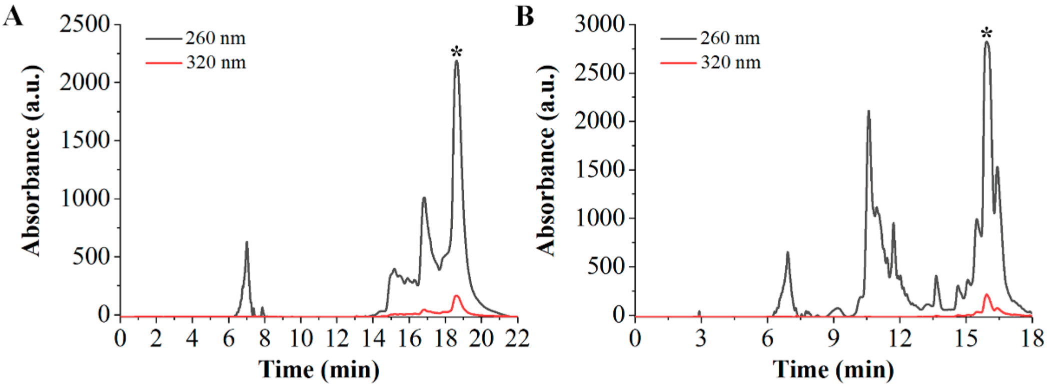

Figure S2. Reverse phase-HPLC spectra of YFNP-1 (A) and YFNP-2 (B) with absorbance at both 260 nm (black) and 320 nm (red). The fraction labeled with "*" was collected.

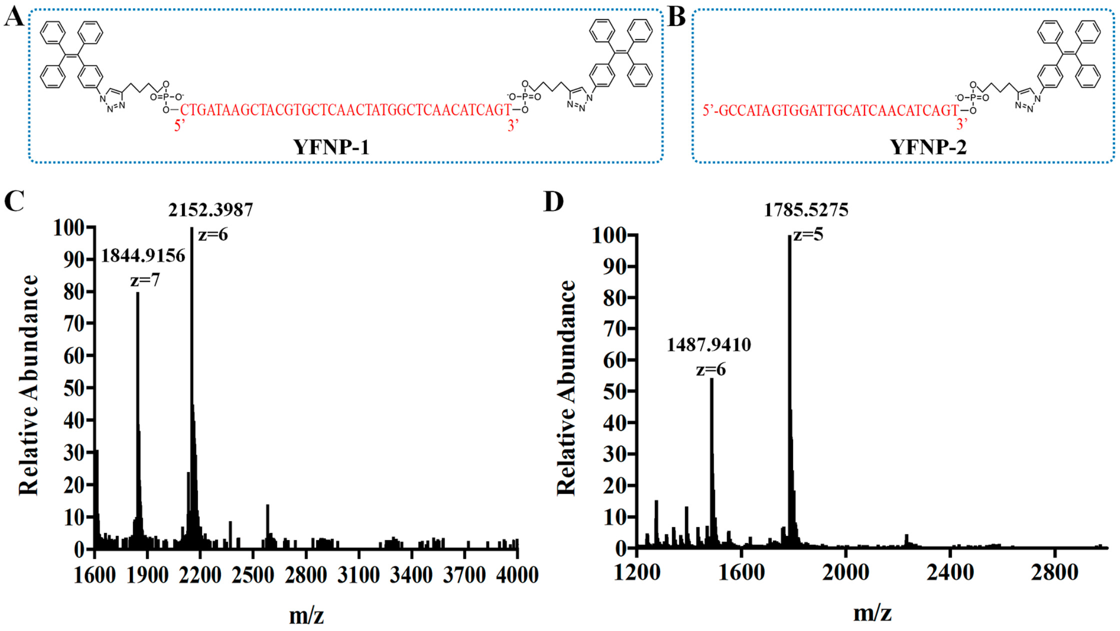

Figure S3. Chemical structure and electrospray ionization (ESI) MS spectrum of YFNP-1 (A and C) and YFNP-2 (B and D). YFNP-1: m/z=2152.3987, z=6 (12918.12 theoretical); YFNP-2: m/z=1785.5275, z=5 (8931.66 theoretical).

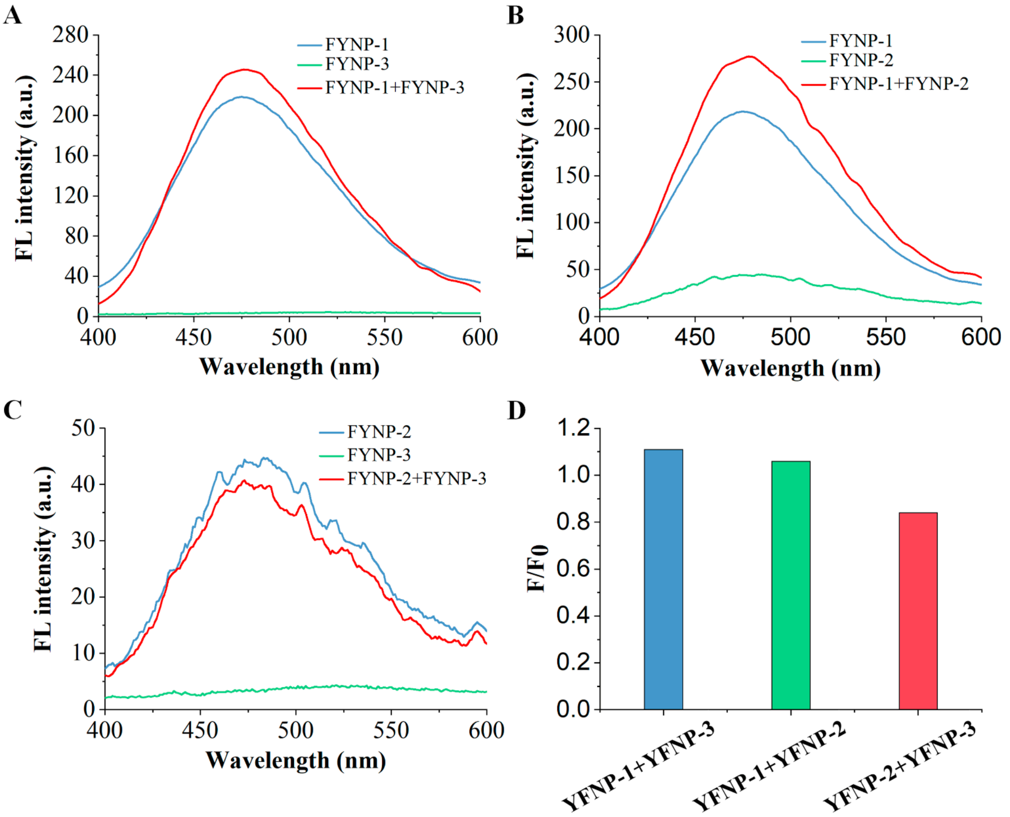

Figure S4. (A-C) The fluorescence spectra of the single-stranded DNA-TPE conjugates (YFNP-1, YFNP-2 and YFNP-3) and their double-stranded hybrids (YFNP-1 + YFNP-

3, YFNP-1 + YFNP-2 and YFNP-2 + YFNP-3) before the addition of target miR-21 in Figure 2B. (D) The AIE effect was evaluated by fluorescence changes before and after the hybridization of the two DNA-TPE conjugates (YFNP-1 + YFNP-3, YFNP-1 + YFNP-2 and YFNP-2 + YFNP-3). F was the fluorescence intensity of the double-stranded hybrids after hybridization of the two DNA-TPE conjugates; F_0 was the sum of the fluorescence intensity of the two DNA-TPE conjugates before their hybridization.

As shown in Figure S3, the single-stranded YFNP-3 showed extremely low fluorescence (Figure S3A) because there was no modification of TPE molecule. The single-stranded YFNP-2 (DNA length = 27 nt) was chemically labelled with only one TPE. By coupling the hydrophilic DNA, the YFNP-2 displayed satisfactory water-solubility, showing a relatively enhanced fluorescence intensity (Figure S3B). However, the single-stranded YFNP-1 (DNA length = 38 nt) with modification of two TPE molecules showed a higher fluorescence compared to the YFNP-2 (Figure S3A). The phenomenon might be attributed to the ssDNA with longer length having more flexible conformation. And the exposed bases would wrap around the hydrophobic TPE and make the hydrophilic phosphate backbones outward to reduce the surface energy, resulting in a large increase in fluorescence, which had also been certified on DNA-TPE conjugates with longer DNA length [43]. Therefore, the double-stranded hybrids (YFNP-1 + YFNP-3 and YFNP-1 + YFNP-2) had a significant fluorescence enhancement after the hybridization (Figure S3A and S3B) since the hybrids had extended sticky ends that modified with more than two TPE molecules. As for the double-stranded hybrids of YFNP-2 and YFNP-3, only one extended sticky end that modified with one TPE molecule, therefore, the hybrids showed a relatively low fluorescence increase (Figure S3C). Besides, the AIE effect was evaluated by detecting fluorescence changes before and after hybridization of the two DNA-TPE conjugates (YFNP-1 + YFNP-3, YFNP-1 + YFNP-2 and YFNP-2 + YFNP-3). As shown in Figure S3D, the fluorescence intensity of the hybrid (YFNP-1 + YFNP-3) is about 1.1 times of the sum of the fluorescence intensity of the single-stranded YFNP-1 and YFNP-3, indicating no occurrence of the AIE effect after the hybridization. And similar results

were found on the hybrids of YFNP-1 and YFNP-2, YFNP-2 and YFNP-3. These results confirmed that the enhanced fluorescence of the hybrids alone without miRNA was originated from the modification of two TPE molecules of YFNP-1, which can be wrapped around by the exposed bases of the extended sticky ends.

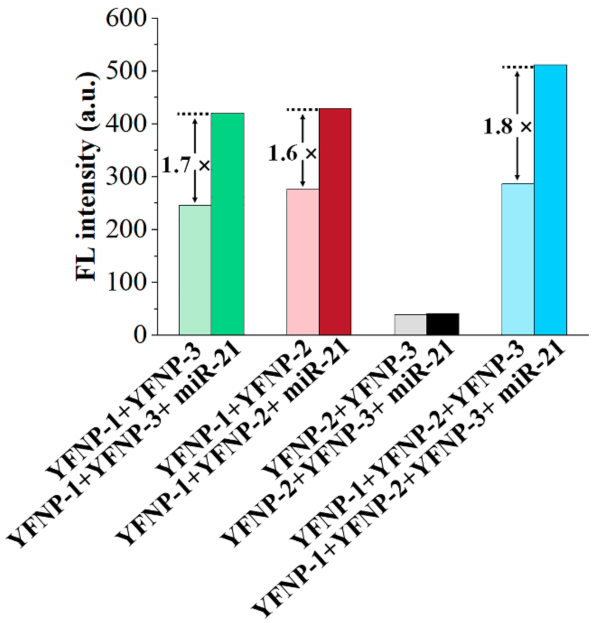

Figure S5. Change of the fluorescence intensity of the assembled YFNP and the double-stranded hybrids (YFNP-1 + YFNP-3, YFNP-1 + YFNP-2 and YFNP-2 + YFNP-3) before and after the addition of target miR-21 in Figure 2B.

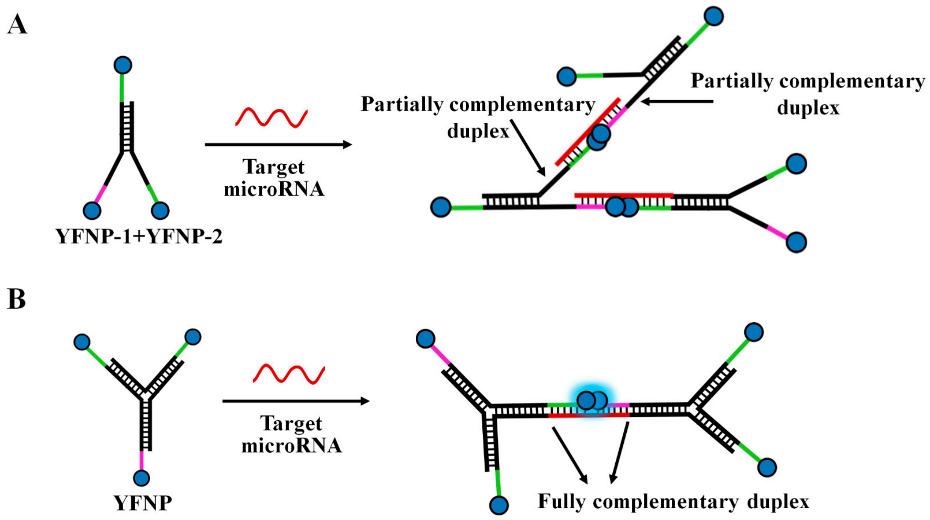

Figure S6. Schematic representation of the YFNP (A) and the hybrid (YFNP-1 + YFNP-2) (B) in response to fully matched target miRNA.

As for the YFNP showed the strongest fluorescence in the presence of miR-21 than that of the double-stranded hybrids (YFNP-1 + YFNP-3, YFNP-1 + YFNP-2 and YFNP-2 + YFNP-3), there are two important reasons. (i) The YFNP and double-stranded hybrid (YFNP-1 and YFNP-2) had three extended sticky ends with three TPE molecules that can recognize miRNA from three directions, while the double-stranded hybrids (YFNP-1 + YFNP-3, YFNP-2 + YFNP-3) only have two and one extended sticky ends for the recognition of the miRNA. Therefore, the YFNP and double-stranded hybrids (YFNP-1 + YFNP-2) showed higher fluorescence in the presence of miR-21 than that of the double-stranded hybrids.

(ii) As for the YFNP and double-stranded hybrid (YFNP-1 and YFNP-2) both had three extended sticky ends with three TPE molecules, however, the environment of the TPE aggregation was different as shown in Figure S5. DNA-AIE based probes have been designed for the detection of nucleic acid based on a structure-dependent light response mechanism [45,46,27]. Lei et al. made a molecular scaffold based on a tetrapod DNA quadruplex (TP-G4) for self-assembly and precise control of the AIE-effect-based fluorescence signal transduction [27]. The AIE effect could be precisely regulated by the distance between the G-quadruplex core and the AIEgens and by altering the quartet number of the G-quadruplex, exhibiting a structure-dependent light response to both tetra- and bimolecular i-motif quadruplex structures. Thus, the conformational change of DNA can be used to induce the AIE effect and detect the nucleic acid. From Figure S5A, partially complementary duplex could be formed after the addition of target miRNA to the hybrid of YFNP-1 and YFNP-2, while fully complementary duplex could be formed after the recognition of YFNP and target (Figure S5B). The fully complementary duplex can provide more confined environment that can induce higher fluorescence because the conformational change is essential for regulating AIE fluorescence. Consequently, the YFNP showed the strongest fluorescence in the presence of miR-21.

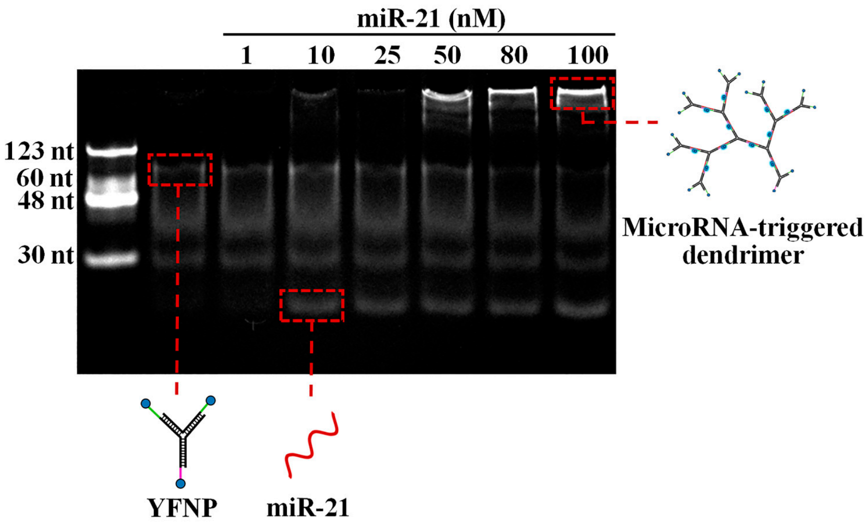

Figure S7. 15% native PAGE characterization of the target-triggered dendrimer responding to various concentrations of miRNA-21 in the presence of the YFNP.

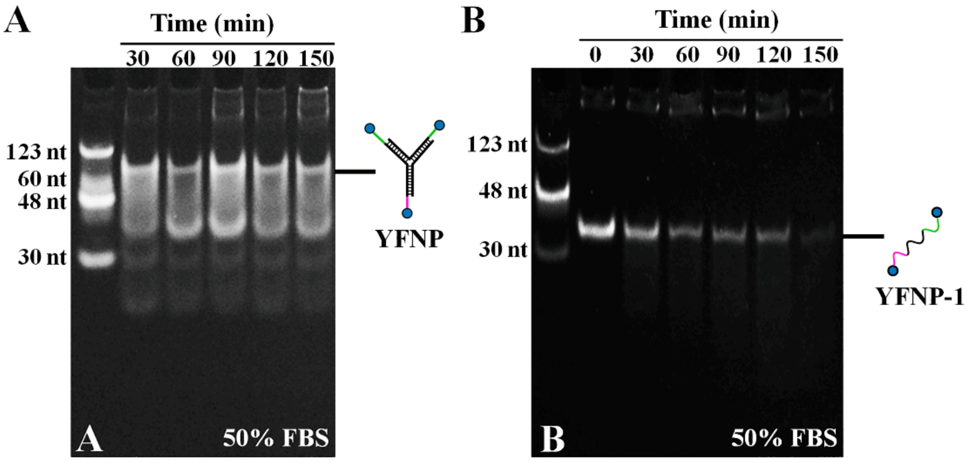

Figure S8. 10% PAGE analysis of the bio-stability of the self-assembled YFNP (A) and the single-stranded YFNP-1 (B) in the presence of 50% FBS.

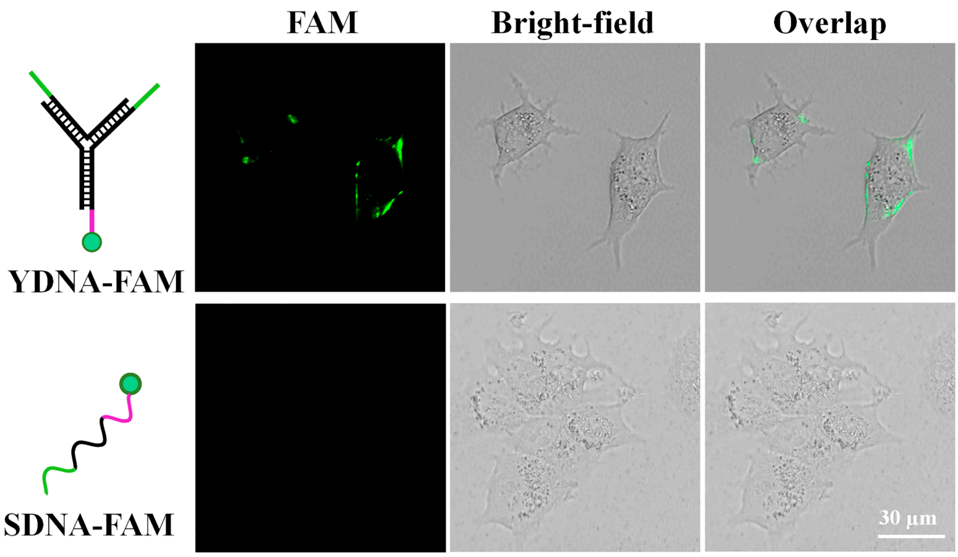

Figure S9. The confocal fluorescence images of MCF-7 cells incubated with YDNA-FAM and SDNA-FAM, respectively.

Table S2. The summary of recent miRNA analytical approaches in comparison with our strategy.

Detection method	Detection limit	Intracellular miRNA imaging	Reference
Exonuclease I-Based fluorescent biosensor	500.0 pM	Unsuitable	31
Colorimetric sensor	3.2 nM	Unsuitable	32
Fluorescent biosensor	14.0 nM	Unsuitable	33
Fluorescent biosensor	98.0 pM	Unsuitable	34
Fluorescent biosensor	200.0 pM	Unsuitable	35
Fluorescent biosensor based on nitrogen-doped carbon dots	29.0 nM	Not mentioned	36
DNA nanowire-based fluorescent biosensor	100.0 pM	Suitable for intracellular miRNA imaging	46
Fluorescent biosensor based on target-catalyzed hairpin assembly	200.0 pM	Suitable for intracellular miRNA imaging	37
**AIE-modified Y-shape DNA nanoprobe-based fluorescent biosensor**	**122.8 pM**	**Suitable for intracellular miRNA imaging**	**This work**

Table S3. Results of recovery analysis by the YFNP-based assay.

Spike miR-21 level (nM)	Mean ± (standard deviation) SD	Average recovery (%)
10	10.94 ± 1.53	109.4
60	57.61 ± 1.07	96.0

Reference:

43. Chen, Z.; Wei, Z.; Xiao, F.; Chao, Z.; Lu, J.; Wang, Z.; Tian, L. The Hydrophobicity of AIE Dye Facilitates DNA Condensation for Carrier-Free Gene Therapy. *Adv. Funct. Mater.* **2022**, *32*, 2207845.
45. Li, Y. Q.; Kwok, R. T. K.; Tang, B. Z.; Liu, B. Specific nucleic acid detection based on fluorescent light-up probe from fluorogens with aggregation-induced emission characteristics. *RSC Adv.* **2013**, *3*, 10135-10138.
46. Zhang, R. Y.; Kwok, R. T. K.; Tang, B. Z.; Liu, B. Hybridization induced fluorescence turn-on of AIEgen-oligonucleotide conjugates for specific DNA detection. *RSC Adv*. **2015**, *5*, 28332-28337.
27. Zhu, L.; Zhou, J.; Xu, G.; Li, C.; Ling, P.; Liu, B.; Ju, H.; Lei, J. DNA quadruplexes as molecular scaffolds for controlled assembly of fluorogens with aggregation-induced emission. *Chem. Sci.* **2018,** *9*, 2559-2566.
31. Ma, C.; Liu, H.; Wu, K.; Chen, M.; Zheng, L.; Wang, J. An Exonuclease I-Based Quencher-Free Fluorescent Method Using DNA Hairpin Probes for Rapid Detection of MicroRNA. *Sensors* **2017**, *17*, 760.
32. Zhao, H.; Qu, Y.; Yuan, F.; Quan, X. A visible and label-free colorimetric sensor for miRNA-21 detection based on peroxidase-like activity of graphene/gold-nanoparticle hybrids. *Anal. Methods* **2016**, *8*, 2005-2012.
33. Oladepo, S. A. Design and Characterization of a Singly Labeled Fluorescent Smart Probe for In Vitro Detection of miR-21. *Appl. Spectrosc.* **2018**, *72*, 79-88.
34. Li, S.; He, K.; Liao, R.; Chen, C.; Chen, X.; Cai, C. An interference-free and label-free sandwich-type magnetic silicon microsphere -rGO-based probe for fluorescence detection of microRNA. *Talanta* **2017**, *174*, 679-683.
35. Hu, X. M.; Li, R. T.; Zhang, M. M.; Wu, K. Y.; Li, H. H.; Huang, N. H.; Sun, B.; Chen, J. X. Phenanthroline-linked berberine dimer and fluorophore-tagged DNA conjugate for the selective detection of microRNA-185: Experimental and molecular docking studies. *Anal. Chim. Acta* **2019**, *1051*, 153-159.
36. Wu, H.; Xu, M.; Chen, Y.; Zhang, H.; Shen, Y.; Tang, Y. A Highly Sensitive and Selective Nano-Fluorescent Probe for Ratiometric and Visual Detection of Oxytetracycline Benefiting from Dual Roles of Nitrogen-Doped Carbon Dots. *Nanomaterials* **2022**, *12*, 4306.
46. Li, Z.; Li, Q.; Wu, Y.; Yuan, K.; Shi, M.; Li, Y.; Meng, H. M.; Li, Z. Multivalent self-assembled nano string lights for tumor-targeted delivery and accelerated biomarker imaging in living cells and in vivo. *Analyst* **2022**, *147*, 811-818.
37. Gu, J.; Qiao, Z.; He, X.; Yu, Y.; Lei, Y.; Tang, J.; Shi, H.; He, D.; Wang, K. Enzyme-free amplified detection of miRNA based on target-catalyzed hairpin assembly and DNA-stabilized fluorescent silver nanoclusters. *Analyst* **2020**, *145*, 5194-5199.
